# Vascular and Myocardial Function in Patients with Type 2 Diabetes and Ischemic Stroke Treated with Dulaglutide or Empagliflozin

**DOI:** 10.3390/medicina62020254

**Published:** 2026-01-25

**Authors:** George Pavlidis, Vasiliki Prentza, Ignatios Ikonomidis, Konstantinos Katogiannis, Aikaterini Kountouri, John Thymis, Eleni Michalopoulou, Loukia Pliouta, Emmanouil Korakas, Maria-Ioanna Stefanou, Lina Palaiodimou, Georgios Tsivgoulis, Vaia Lambadiari

**Affiliations:** 1 4th Department of Internal Medicine, Attikon University Hospital, Medical School, National and Kapodistrian University of Athens, 12462 Athens, Greece; 2 2nd Department of Cardiology, Attikon University Hospital, Medical School, National and Kapodistrian University of Athens, 12462 Athens, Greece; prentzavasiliki@gmail.com (V.P.); ignoik@gmail.com (I.I.); kenndj89@gmail.com (K.K.); johnythg@gmail.com (J.T.); elenimixa91@gmail.com (E.M.); 3 2nd Department of Internal Medicine, Research Unit and Diabetes Center, Medical School, National and Kapodistrian University of Athens, 12462 Athens, Greece; katerinak90@hotmail.com (A.K.); plioutaloukia@gmail.com (L.P.); mankor-th@hotmail.com (E.K.); vlambad@otenet.gr (V.L.); 4 2nd Department of Neurology, Attikon University Hospital, Medical School, National and Kapodistrian University of Athens, 12462 Athens, Greece; marianna421@hotmail.co.uk (M.-I.S.); lina_palaiodimou@yahoo.gr (L.P.); tsivgoulisgiorg@yahoo.gr (G.T.)

**Keywords:** type 2 diabetes, ischemic stroke, glucagon-like peptide-1 receptor agonists, sodium-glucose cotransporter 2 inhibitors, endothelial glycocalyx, arterial stiffness, left ventricular myocardial strain

## Abstract

*Background and Objectives:* Patients with type 2 diabetes mellitus (T2DM) and ischemic stroke present with endothelial, vascular and left ventricular (LV) myocardial dysfunction. We investigated the effects of treatment with either glucagon-like peptide-1 receptor agonists (GLP-1RA) or sodium-glucose contrasporter-2 inhibitors (SGLT-2i) on endothelial glycocalyx, arterial stiffness, and LV myocardial strain in patients with metformin-treated T2DM and a prior ischemic stroke. *Materials and Methods:* A total of 54 consecutive patients with T2DM and ischemic stroke who attended a cardiometabolic outpatient clinic in Athens, Greece, and received either GLP-1RA (dulaglutide; n = 27) or SGLT-2i (empagliflozin; n = 27) were enrolled in the study. We measured the perfused boundary region (PBR) of the sublingual microvessels, a marker of glycocalyx thickness, as well as carotid-femoral pulse wave velocity (PWV) and LV global longitudinal strain (GLS), at baseline and at 4 and 12 months of treatment. *Results:* Twelve months after treatment, all patients had reduced glycosylated hemoglobin and body mass index (BMI) (*p* < 0.001). Patients treated with dulaglutide showed a greater reduction in BMI (−11.8% vs. −4.8%, *p* < 0.001) compared to those treated with empagliflozin. Compared to baseline, all patients had reduced PBR, PWV and GLS (*p* < 0.001) after 12 months of treatment. However, empagliflozin presented a greater decrease in PWV (−14% vs. −10.9%, *p* = 0.041), while dulaglutide resulted in a greater increase in GLS (14.7% vs. 8.3%, *p* = 0.024) compared to empagliflozin. In all patients, the reduction in PBR at 12 months was correlated with a decrease in PWV and with an increase in GLS (*p* < 0.05). *Conclusions:* Both dulaglutide and empagliflozin improve cardiovascular function in T2DM patients with ischemic stroke. Dulaglutide appears to be more effective in the improvement of LV myocardial strain, whereas empagliflozin is more effective in reducing arterial stiffness.

## 1. Introduction

Diabetes mellitus (DM) is an independent risk factor for the development of acute cerebrovascular events, such as ischemic stroke. Disruption of vascular endothelial integrity appears to be the primary target in DM [1], resulting in microangiopathy and macroangiopathy, including ischemic stroke [2]. The main mechanisms involved in endothelial dysfunction in DM are chronic hyperglycemia, atherogenic dyslipidemia, and insulin resistance [3].

Endothelial glycocalyx is a complex layer of proteoglycans and glycoproteins that covers the intraluminal surface of the endothelium [4]. Studies demonstrate that this layer plays an important role in vascular permeability, the adhesion of white blood cells and platelets, and the mediation of shear stress [5,6,7,8]. Vascular regions with an impaired endothelial glycocalyx are more prone to proinflammatory and atherosclerotic lesions [9]. New imaging techniques allow the non-invasive assessment of glycocalyx thickness in the sublingual capillary network using a dedicated camera [4].

In addition, arterial stiffness is exacerbated by pathological conditions, such as DM [10]. Increased pulse wave velocity (PWV) and augmentation index (AIx) are indicators of arterial stiffness and reflected waves, respectively [11,12]. In subjects with DM, increased arterial stiffness [13] is associated with the formation of advanced glycation end products and subsequent cross-linking polymerization with collagen.

The assessment of left ventricular (LV) myocardial strain using two-dimensional echocardiographic imaging is considered a highly sensitive method for assessing LV myocardial performance. The LV global longitudinal strain (GLS) determined by this method can detect subclinical LV myocardial dysfunction, which has particular prognostic value for DM patients [14].

Newer antidiabetic agents, such as glycogen-like peptide-1 receptor agonists (GLP-1RA) and sodium-glucose cotransporter type 2 inhibitors (SGLT-2i), have potential anti-inflammatory properties associated with intrinsic pharmacological class actions beyond glycemic control [15,16]. More specifically, treatment with GLP-1RA and SGLT-2i offers significant improvement in endothelial function, arterial stiffness, and LV myocardial function due to their favorable cardiovascular effects [17,18]. Additionally, in the context of primary prevention, the improvement in glycemic control after treatment with GLP-1RA is associated with a reduction in the risk of ischemic stroke in individuals with DM [19].

In light of the aforementioned evidence, the aim of the present study is to investigate the effects of dulaglutide (GLP-1RA) and empagliflozin (SGLT-2i) on endothelial glycocalyx, arterial stiffness, and LV myocardial function in patients with type 2 diabetes mellitus (T2DM) and ischemic stroke at 4 and 12 months of treatment.

## 2. Materials and Methods

### 2.1. Study Design

The present study was a single-center observational cohort study. A total of 138 consecutive metformin-treated patients with type 2 diabetes mellitus (T2DM) and ischemic stroke who attended the cardiometabolic outpatient clinic were enrolled by their attending physicians (I.I. and V.L.). Of those, 71 patients received dulaglutide (0.75 mg once weekly for the first month and 1.5 mg once weekly thereafter) and 67 received 25 mg oral empagliflozin once daily as add-on to metformin for 12 months.

The inclusion criteria were male or female patients aged 18 to 75 years with T2DM and a history of ischemic stroke (atherosclerotic, cardioembolic or lacunar) more than 6 months prior to enrollment. The exclusion criteria were haemorrhagic stroke, chronic kidney disease (estimated glomerular filtration rate < 60 mL/min/m^2^ for a period of at least 90 days), peripheral artery disease, retinopathy, chronic inflammatory disease, or active malignancies. The recruitment took place between June 2022 and December 2024. All participants underwent clinical, vascular, and echocardiography examination at 4 and 12 months after inclusion in the study. None of the female participants was receiving hormone replacement treatment.

The investigation was conducted in accordance with the ethical principles outlined in the Declaration of Helsinki and its later amendments. The study protocol was approved by the Institutional Ethics Committee. In addition, all participants provided written informed consent prior to inclusion.

### 2.2. Endothelial Glycocalyx Measurement

A rapid, non-invasive method to assess the endothelial glycocalyx is to measure the perfused boundary region (PBR) of sublingual vessels (5–25 μm in diameter) using a special high-resolution camera with the Sideview Darkfield Imaging technique (Microscan, Glycocheck, Microvascular Health Solutions Inc., Salt Lake City, UT, USA) [20]. An increased PBR value indicates deeper penetration of red blood cells into the glycocalyx, suggesting a loss of glycocalyx barrier properties and a reduction in glycocalyx thickness. Measurement of endothelial glycocalyx thickness using the Sideview Darkfield Imaging technique provides measurements at multiple sample sites (>3000 vascular segments of the sublingual microvasculature), which have very good reproducibility [21].

### 2.3. Arterial Stiffness Measurement

Carotid-femoral pulse wave velocity (PWV) and central aortic systolic blood pressure (SBP) were estimated through tonometry (Complior, Alam Medical, Vincennes, France). The assessment of PWV involved capturing carotid and femoral arterial waveforms using two non-invasive sensors, while the physical distance between these two arterial sites was measured using a standard tape measure. A PWV value lower than 10 m/s was considered within the normal range [22].

### 2.4. Echocardiographic Assessment of Left Ventricular Myocardial Strain

Left ventricular global longitudinal strain (LV GLS) was measured using a 17-segment model and specialized strain analysis software (EchoPac PC 206; GE Healthcare, Horten, Norway) according to a previously reported methodology [17]. Normal GLS is reported as −22.5 ± 2.7% [23].

### 2.5. Statistical Analysis

The primary outcome of the present study was the determination of the changes in PBR, and, therefore, for the sample size calculation of the two study groups, we performed an a priori power calculation based on an initial pilot cohort. The pilot sample included 20 patients recruited into the study and assigned to two treatment groups [dulaglutide (GLP-1RA) and empagliflozin (SGLT-2i)]. The percentage change in PBR score (ΔPBR%) at 4 months was calculated for the purpose of power analysis (dulaglutide: 6% and empagliflozin: 3.6%, respectively). To detect a relevant reduction in PBR with a two-sided α = 0.05 (to control the risk of type I error), a power of 0.80, and a SD of 3.3, it was necessary to include at least n = 24 patients in each group. To accommodate attrition, we inflated this number by 15% and rounded it up to 30 per group. Because we prespecified 1:1 propensity score matching (PSM) and anticipated ≈40–50% loss during PSM, we targeted at least 110 patients before matching (~55 patients per group).

We implemented PSM to create comparable groups and avoid any potential confounding. Propensity scores were constructed utilizing a probit regression model that included age, sex, body mass index (BMI), glycosylated hemoglobin (HbA1c), and total cholesterol at baseline. We applied nearest-neighbor matching with a 1:1 ratio without replacement and a caliper of 0.2 to match participants in the GLP-1RA group to those in the SGLT-2i group. After matching, standardized mean differences were used to estimate covariate balance, with a standardized mean difference <0.1 considered indicative of a sufficiently balanced model.

The Kolmogorov–Smirnov and Shapiro–Wilk normality tests were performed to determine the distribution. Continuous variables were presented as mean ± standard deviation in the case of normal distribution or as median with interquartile range in the case of non-normally distributed variables. Differences in continuous variables were calculated by the Student’s t-test and the Mann–Whitney U test, as appropriate. Categorical variables were expressed as counts and percentages (%) and were analyzed using the chi-square test. Analysis of variance (ANOVA) for repeated measurements was applied (a) for the measurements of the examined markers at baseline and at 4 and 12 months of antidiabetic treatment, which was considered as a within-subject factor, and (b) for the effects of treatment (dulaglutide and empagliflozin) as a between-subject factor. No post hoc correction for multiple testing was applied to the repeated measurements ANOVA. The time-by-treatment group interaction term was calculated. Age, sex, hypertension, hyperlipidemia, concomitant medications, fasting glucose, HbA1c, BMI, mean blood pressure and their changes at 4 and 12 months of treatment were included as covariates in our model to control for potential confounders. The F and *p* values of the interaction between time of measurement of the examined markers and the type of treatment were evaluated. Moreover, the F and the corresponding *p* values of the comparison between treatments were calculated. Subgroup and correlation analyses were carried out as exploratory analyses and should be interpreted accordingly. Data analysis was conducted using the Statistical Package for Social Sciences (IBM SPSS Statistics for Windows, Version 28.0. Armonk, NY, USA). All statistical tests were performed as two-tailed, and a *p* value < 0.05 was considered statistically significant.

## 3. Results

Out of the 138 enrolled subjects, 25 patients were excluded because of the above exclusion criteria. The remaining 113 participants (58 in the GLP-1RA group and 55 in the SGLT-2i group) were included in the initial analysis. Based on our sample power calculation for this study indicating that at least 24 patients were required in each treatment group, patients under metformin and GLP-1RA were 1:1 matched to patients under metformin and SGLT-2i, yielding 30 matched pairs by propensity score matching analysis. The matching using propensity scores was applied in order to have two study groups (dulaglutide and empagliflozin) in balance for age, sex, BMI, HbA1c, and total cholesterol at baseline.

**Figure 1:**
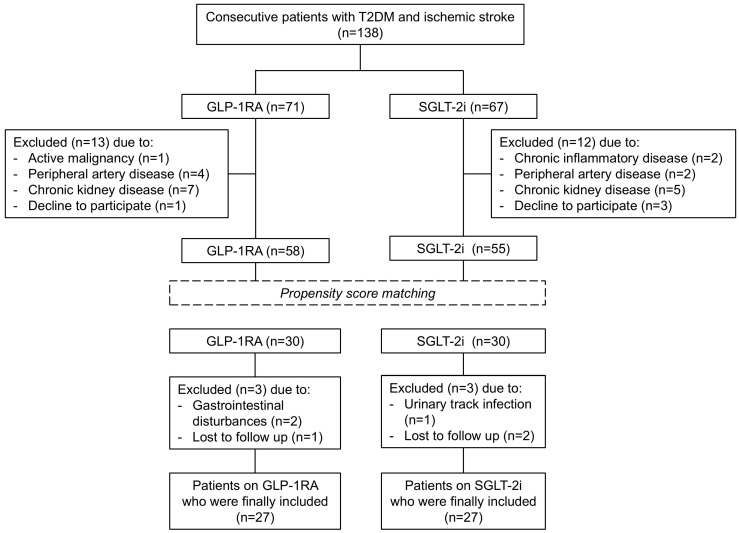
Flowchart of the study cohort. T2DM, type 2 diabetes mellitus; GLP-1RA, glucagon-like peptide-1 receptor agonists; SGLT-2i, sodium-glucose contrasporter-2 inhibitors.

A total of 6 subjects did not complete the study protocol. More specifically, in the dulaglutide group, 2 participants discontinued due to gastrointestinal disturbances (nausea, vomiting, or diarrhea) and 1 subject was lost to follow-up. In the empagliflozin group, 1 participant discontinued owing to urinary tract infection, and 2 subjects were lost to follow-up. Thus, 54 subjects (27 subjects in the dulaglutide group and 27 in the empagliflozin group) were included in the final analysis (Figure 1).

### 3.1. Baseline Characteristics

Demographic and clinical characteristics of the matched population are summarized in Table 1. The mean age (SD) of patients was 66.4 (6.9) years, and the majority were males (51.9%). In addition, 20.4% of the participants had overt coronary artery disease. Age, sex, duration of T2DM, HbA1c, BMI, cardiovascular risk factors, and medication use were similar between the two treatment groups (*p* > 0.05 for all comparisons; Table 1). Furthermore, there were no statistically significant between-group differences at baseline in metabolic, vascular, and LV myocardial markers (*p* > 0.05; Table 2 and Table 3).

### 3.2. Effect of Treatment on Metabolic Markers

All patients had reduced fasting glucose, HbA1c, and BMI at 4 months and 12 months (*p* < 0.001 for all comparisons). However, there was a significant interaction of time with the type of treatment and BMI (F = 6.647, *p* < 0.001). Patients who were treated with dulaglutide showed a greater reduction in BMI at 4 months (−7.5% vs. −3.5%, *p* = 0.004) and 12 months (−11.8% vs. −4.8%, *p* < 0.001) compared to those under empagliflozin. Moreover, total cholesterol, low-density lipoprotein cholesterol, and triglycerides were decreased in the whole study population at the same time points (*p* < 0.05; Table 2).

### 3.3. Effect of Treatment on Vascular and Myocardial Markers

**Figure 2:**
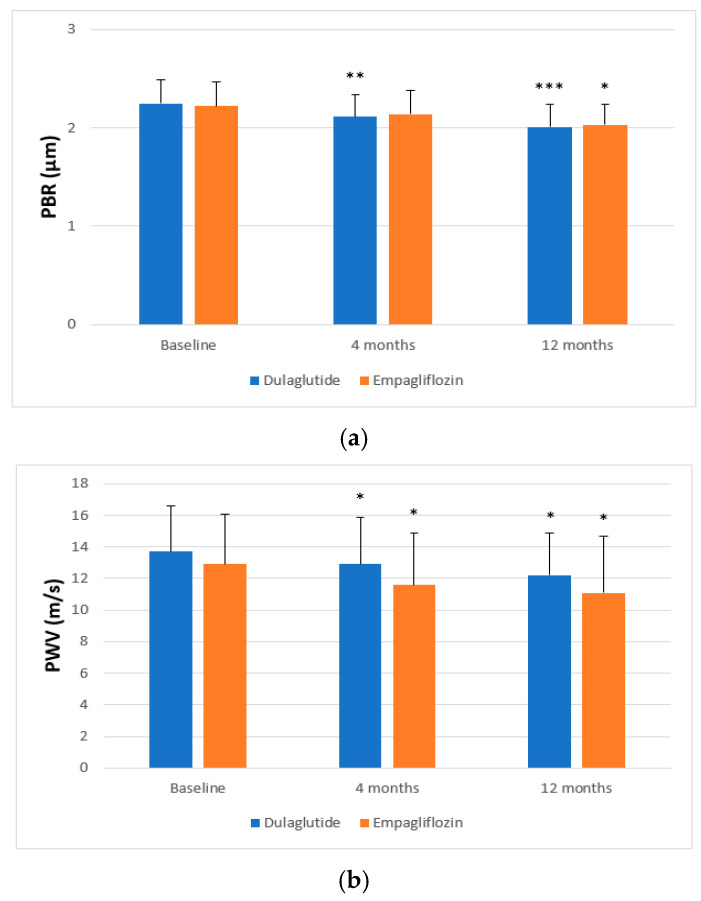

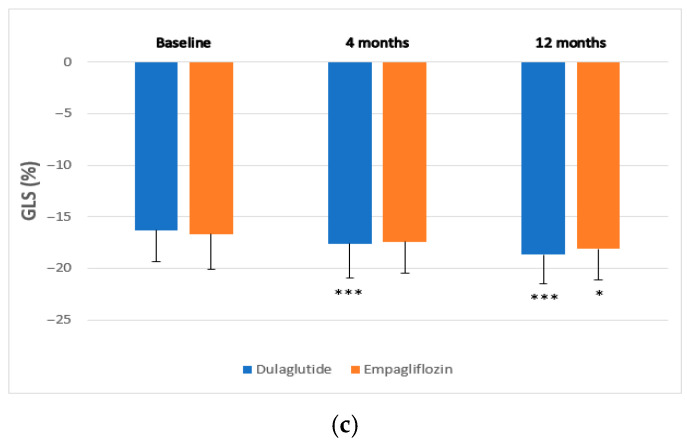
Changes in (**a**) perfused boundary region (PBR) of the sublingual microvessels (5–25 μm), (**b**) pulse wave velocity (PWV), and (**c**) left ventricular global longitudinal myocardial strain (GLS) after 4 and 12 months of treatment with dulaglutide or empagliflozin. * *p* < 0.05, ** *p* < 0.01, *** *p* < 0.001 for comparisons of mean values at 4 and 12 months with baseline mean values.

In the overall study population, a significant decrease in vascular markers was observed. More specifically, PBR (*p* = 0.007 and *p* < 0.001; Figure 2a), SBP (*p* = 0.044 and *p* < 0.001), central SBP (*p* = 0.012 and *p* < 0.001), PWV (*p* = 0.003 and *p* < 0.001), and AIx (*p* < 0.001) were reduced in all patients at 4 and 12 months, respectively (Table 3). Notably, there was a significant interaction between follow-up time and the type of treatment for PWV (F = 4.154, *p* = 0.045) in a model including age, sex, hypertension, hyperlipidemia, concomitant medications, fasting glucose, HbA1c, BMI, mean blood pressure, and their changes at 4 and 12 months. Patients treated with empagliflozin presented a greater decrease in PWV at 4 months (−10.1% vs. −5.8%, *p* = 0.037) and 12 months (−14% vs. −10.9%, *p* = 0.041) in comparison with those treated with dulaglutide (Figure 2b). There were no statistically significant differences in the improvement of PBR, brachial and central SBP, and AIx between the two study groups post-treatment (*p* > 0.05; Table 3).

Similarly, in relation to echocardiographic markers, all participants had increased GLS at the 4-month and 12-month follow-up (*p* < 0.001). There was a significant interaction between time and the type of treatment with regard to GLS (F = 9.409, *p* = 0.003). In particular, dulaglutide administration resulted in a greater increase in GLS at 4 months (8% vs. 4.2%, *p* = 0.038) and at 12 months of treatment (14.7% vs. 8.3%, *p* = 0.024) compared with empagliflozin (Figure 2c).

### 3.4. Association of Metabolic, Vascular, and Myocardial Markers

In the whole study population, baseline HbA1c was correlated with PBR (r = 0.329, *p* = 0.028), PWV (r = 0.471, *p* = 0.001), and GLS (r = 0.306, *p* = 0.044).

In all patients, the percentage reduction in BMI at 4 and 12 months of treatment was associated with the decrease in PBR (r = 0.291, *p* = 0.047 and r = 0.350, *p* = 0.039, respectively), PWV (r = 0.305, *p* = 0.042 and r = 0.572, *p* = 0.006), and central SBP (r = 0.435, *p* = 0.033 and r = 0.539, *p* = 0.024). Moreover, the percentage decrease in HbA1c at 4 and 12 months was directly correlated with the reduction in PBR (r = 0.341, *p* = 0.035 and r = 0.524, *p* = 0.004), PWV (r = 0.621, *p* = 0.035 and r = 0.446, *p* = 0.020) and with the improvement in GLS (r = 0.309, *p* = 0.045 and r = 0.392, *p* = 0.023).

In addition, the percentage reduction in PBR in all patients at 4 and 12 months of treatment was associated with a respective decrease in PWV (r = 0.325, *p* = 0.041 and r = 0.411, *p* = 0.022) and AIx (r = 0.308, *p* = 0.034 and r = 0.341, *p* = 0.032) and with an increase in GLS (r = 0.418, *p* = 0.012 and r = 0.399, *p* = 0.027).

## 4. Discussion

In the present study, we found that treatment with either dulaglutide or empagliflozin was associated with a significant reduction in fasting glucose, HbA1c, and BMI in all study patients after 12 months of treatment. However, patients treated with dulaglutide showed a greater reduction in BMI compared to those treated with empagliflozin. On the other hand, empagliflozin was associated with a greater reduction in PWV, while the improvement in GLS appeared to be greater with dulaglutide treatment compared with empagliflozin. Finally, in all patients, the improvement in endothelial glycocalyx, as assessed by PBR measurement, was associated with improvement in arterial stiffness and left ventricular myocardial strain.

Diabetes mellitus is a metabolic disease accompanied by vascular complications related to endothelial dysfunction due to disturbances in the structure of the endothelial glycocalyx [24,25]. The disruption of glycocalyx structure in patients with DM is due to multiple mechanisms, including interaction of glycocalyx glycoproteins with glucose [24], changes in heparan sulfate biosynthesis [26], increased plasma levels of hyaluronic acid, hyaluronidase [27], and inflammatory mediators, which in some cases can induce the formation of intercellular holes, increasing vascular permeability [28,29], as well as reduced production of vascular endothelial growth factor [30]. GLP-1RA have been shown to improve endothelial function and reduce inflammation and atherosclerosis [31]. Similarly, SGLT-2i have been demonstrated to protect the endothelial glycocalyx [17]. Interestingly, patients with T2DM who received empagliflozin within a short period after acute myocardial infarction showed a significant improvement in endothelial glycocalyx thickness, flow-mediated dilation of the brachial artery, as well as improved circulating endothelial-related markers [32]. Measurement of PBR, an indicator of glycocalyx thickness [20] in our study, showed a significant reduction in PBR in the whole study population during the follow-up period, while no statistically significant difference in PBR was detected between dulaglutide and empagliflozin.

In the current study, significant improvement in arterial stiffness, as expressed by the reduction in PWV and AIx, was observed in all patients at 4 and 12 months. There was no statistically significant difference in the improvement in AIx between the two groups after treatment. However, patients who were treated with empagliflozin experienced a greater reduction in PWV post-treatment compared with those on dulaglutide. Indeed, SGLT-2i cause glycosuria, osmotic diuresis, natriuresis, a decrease in interstitial fluid, a reduction in blood pressure, and ultimately a decrease in vascular resistance and arterial stiffness [33,34].

Several studies have shown that GLP-1RA exert favorable effects on SBP and BMI, while SGLT-2i cause a greater reduction in SBP [33]. In all patients, a significant reduction in SBP and central SBP was observed. No significant differences were observed in the improvement in brachial and central SBP between the two study groups after treatment. Additionally, in both study groups, the percentage reduction in central SBP was associated with the reduction in BMI at 4 and 12 months of treatment, indicating the beneficial effects of weight loss on blood pressure.

In our study, all participants had increased GLS at 4-month and 12-month follow-up examinations. A recent study showed that LV GLS is an independent predictor of all-cause death and recurrent ischemic stroke in patients with acute ischemic stroke, demonstrating its prognostic value regardless of traditional risk factors. In this study, an LV GLS of 18% was the optimal cutoff [35]. Even a modest improvement in GLS, as observed in the present study, appears to be clinically significant in patients without overt heart failure. Interestingly, in the Copenhagen City Heart Study, which included 1296 participants in a low-risk general population, each 1% deterioration in LV GLS was independently associated with a 12% increase in the risk of the composite endpoint (heart failure, acute myocardial infarction, or cardiovascular death) after a mean follow-up of 11 years [36]. Conversely, in patients with psoriasis, a GLS absolute increase post-treatment with a cutoff value of a GLS change of ≥1.44% was indicative of higher major adverse cardiovascular events-free survival over a 4-year follow-up period [37]. In the current study, we found that dulaglutide administration was associated with a greater increase in GLS at the same time points compared with empagliflozin. Several studies have shown that GLP-1RA improve ventricular contractility, enhance glucose uptake by myocardial cells, improve left ventricular deformation, and exert cytoprotective and metabolic effects on the myocardium [38,39]. On the other hand, the cardioprotective effects of SGLT-2i appear to be related to their beneficial effects on hemodynamic parameters [17,33,34]. Accumulating data indicates that SGLT-2i may inhibit Na+/H+ exchange and lead to lower intracellular Na^+^ and Ca^2+^ concentrations while increasing mitochondrial Ca^2+^ concentrations in rat and rabbit myocardial cells [40]. In addition, SGLT-2i increase the utilization of ketone bodies by myocardial cells [41], which improves myocardial contractility and ejection fraction [42]. Recent studies have shown that SGLT-2i have a beneficial effect on left ventricular mass and diastolic function in DM patients with coronary artery disease [43,44]. Several studies have demonstrated that the addition of SGLT-2i to the optimal medical therapy was associated with improvement in LV systolic function, as assessed by GLS and myocardial work index, regardless of the presence of heart failure (HF), especially in those with HF with reduced ejection fraction (HFrEF) [45,46,47]. Importantly, SGLT-2i administration in patients with a long history of HFrEF was related to a significant improvement in right ventricular systolic function [48,49].

All patients in the present study were diagnosed with T2DM and had a history of ischemic stroke. With respect to secondary stroke prevention, it should be noted that evidence from cardiovascular outcome trials and meta-analyses indicates the superiority of GLP-1RA over SGLT-2i in reducing stroke recurrence [19,50,51], with clinical practice guidelines favoring GLP-1RA for secondary stroke prevention [52]. Nevertheless, accruing real-world data report improved stroke outcomes with SGLT-2i [53], while a previous meta-analysis of randomized clinical studies suggested differential effects by stroke subtype, with SGLT-2i demonstrating neutral effects on the risk of ischemic stroke but a significant 50% reduction in hemorrhagic stroke risk compared with placebo [54]. The findings of the present analysis could thus have translational implications for further research, raising the hypothesis that discordant effects may be observed by medication class across stroke subtypes. In this regard, the findings of reduced arterial stiffness with empagliflozin could support the potential of SGLT-2i in treating hemorrhagic, large-artery atherosclerosis or lacunar ischemic stroke subtypes, all characterized by increased arterial stiffness [55,56], a hypothesis that warrants further evaluation in well-designed and adequately powered trials.

Certain limitations should be acknowledged. First, the relatively small number of patients is a limitation of this study, necessitating larger multicenter trials for independent confirmation of the present findings. Second, the study cohort comprised a very specific population, consisting of T2DM patients with prior ischemic stroke. Thus, our findings may not be generalizable to T2DM patients without stroke, primary prevention populations, or patients with established heart failure. Third, patients with advanced diabetic complications, such as peripheral artery disease and diabetic retinopathy, were excluded, which may limit the generalizability of the findings. Fourth, patients were not randomized to treatment groups based on the study design; however, propensity score matching was implemented to minimize baseline differences and mitigate potential selection bias. Fifth, although all analyses adjusted for the changes in BMI and HbA1c, the possibility that vascular and myocardial improvements are mediated by metabolic improvement rather than direct drug-specific actions cannot be fully excluded. On the other hand, the 12-month follow-up period is considered adequate to allow for a robust evaluation of treatment effects on the studied outcomes [17]. In contrast to previous studies that evaluated similar endpoints [17,18], this study is the first to independently evaluate the effects of dulaglutide and empagliflozin on endothelial, vascular, and myocardial function in patients with T2DM and ischemic stroke and directly compare the two classes of antidiabetic therapy.

## 5. Conclusions

Twelve-month treatment with dulaglutide or empagliflozin was associated with improved endothelial glycocalyx, vascular function, and LV myocardial strain in patients with T2DM and a prior ischemic stroke. Dulaglutide appears to be more effective in improving LV myocardial function, likely due to the direct actions of this drug class on the myocardium, whereas empagliflozin appears more effective in reducing arterial stiffness owing to its favorable hemodynamic actions. Overall, these findings highlight the beneficial effects of modern antidiabetic agents on the cardiovascular system in diabetic subjects who have experienced an ischemic stroke. Larger, randomized and long-term clinical trials are warranted to confirm and expand the findings of the present study.

## Figures and Tables

**Table 1:** Demographic and clinical characteristics of the study population.

	All Patients (n = 54)	Dulaglutide (n = 27)	Empagliflozin (n = 27)	*p* Value
Age (years)	66.4 ± 6.9	65.7 ± 6.5	67.1 ± 7.2	0.460
Sex (male), n (%)	28 (51.9)	15 (55.6)	13 (48.1)	0.583
Duration of DM	10 (2.75–16.25)	11 (2–17)	10 (3–16)	0.660
HbA1c (%)	7.85 ± 0.61	7.97 ± 0.67	7.73 ± 0.53	0.443
BMI (kg/m^2^)	31.8 ± 4.4	32.2 ± 4.5	31.3 ± 4.2	0.392
CAD, n (%)	11 (20.4)	6 (22.2)	5 (18.5)	0.735
Risk factors, n (%)				
Hypertension	41 (75.9)	22 (81.5)	19 (70.4)	0.340
Hyperlipidemia	54 (100)	27 (100)	27 (100)	1.000
Current smoker	21 (38.9)	10 (37)	11 (40.7)	0.780
Family history CAD	16 (29.6)	9 (33.3)	7 (25.9)	0.551
Medications, n (%)				
ACEi/ARBs	37 (68.5)	18 (66.7)	19 (70.4)	0.770
CCB	23 (42.6)	13 (48.1)	10 (37)	0.409
β-Blockers	24 (44.4)	14 (51.9)	10 (37)	0.273
Diuretics	26 (48.2)	12 (44.4)	14 (51.9)	0.586
MRA	1 (1.9)	0 (0)	1 (3.7)	0.313
Statins	54 (100)	27 (100)	27 (100)	1.000
Fibrates	4 (7.4)	3 (11.1)	1 (3.7)	0.299
Antiplatelets	54 (100)	27 (100)	27 (100)	1.000

Data are expressed as mean ± standard deviation, median (first quartile–third quartile), or number (%). DM, diabetes mellitus; HbA1c, glycosylated hemoglobin; BMI, body mass index; CAD, coronary artery disease; ACEi, angiotensin-converting enzyme inhibitors; ARBs, angiotensin receptor blockers; CCB, calcium channel blockers; MRA, mineralocorticoid receptor antagonists.

**Table 2:** Effects of dulaglutide versus empagliflozin on metabolic markers during the study period.

	All Patients (n = 54)	Dulaglutide (n = 27)	Empagliflozin (n = 27)
Fasting glucose (mg/dL)			
Baseline	179.4 ± 42.4	180.4 ± 43.2	178.3 ± 41.6
4 months	139.7 ± 29.1 ^†††^	134.2 ± 28.7 ^†††^	145.2 ± 29.5 ^†††^
12 months	121.5 ± 20.2 ^†††^	119.7 ± 21.7 ^†††^	123.3 ± 18.7 ^†††^
HbA1c (%)			
Baseline	7.88 ± 0.61	7.97 ± 0.67	7.78 ± 0.53
4 months	6.98 ± 0.58 ^†††^	6.94 ± 0.71 ^†††^	7.02 ± 0.45 ^†††^
12 months	6.75 ± 0.37 ^†††^	6.57 ± 0.42 ^†††^	6.94 ± 0.32 ^†††^
BMI (kg/m^2^)			
Baseline	31.8 ± 4.4	32.2 ± 4.5	31.3 ± 4.2
4 months	30.1 ± 3.9 ^†††^	29.8 ± 4.1 ^†††^	30.2 ± 3.6 ^†††^
12 months	29.1 ± 3.8 ^†††^	28.4 ± 3.7 ^†††^	29.8 ± 3.8 ^††,^*
Total cholesterol (mg/dL)			
Baseline	174.6 ± 48.3	178.2 ± 52	170.9 ± 44.5
4 months	157.9 ± 43.6 ^††^	160.1 ± 48.5 ^†^	155.8 ± 38.7 ^†^
12 months	154.1 ± 37.3 ^†††^	151.7 ± 34.2 ^†^	156.3 ± 40.3
LDL-C (mg/dL)			
Baseline	101.1 ± 32.2	103.3 ± 34.9	98.8 ± 29.4
4 months	89.3 ± 34.4 ^††^	89.6 ± 36.6 ^†^	88.9 ± 32.2 ^†^
12 months	85.7 ± 32.5 ^†^	81.2 ± 31.4 ^†^	90.2 ± 33.5
HDL-C (mg/dL)			
Baseline	46.9 ± 11.3	46.4 ± 10	47.3 ± 12.5
4 months	47.3 ± 11.2	46.2 ± 9.5	48.3 ± 12.8
12 months	48.5 ± 11.2	47.8 ± 10.9	49.2 ± 11.4
Triglycerides (mg/dL)			
Baseline	153.7 ± 46.8	159.1 ± 49.5	148.3 ± 44.1
4 months	140.2 ± 39.1 ^†^	140.8 ± 37.3 ^†^	139.6 ± 40.8
12 months	122.9 ± 27.8 ^††^	123.9 ± 26.4 ^†^	121.8 ± 29.3 ^†^

Data are presented as mean values ± standard deviation. HbA1c, glycosylated hemoglobin; BMI, body mass index; LDL-C, low-density lipoprotein cholesterol; HDL-C, high-density lipoprotein cholesterol. * *p* < 0.001 for time × treatment interaction obtained by repeated measures ANOVA. ^†^ *p* < 0.05, ^††^ *p* < 0.01, ^†††^ *p* < 0.001 for comparisons of 4 or 12 months vs. baseline.

**Table 3:** Effects of dulaglutide versus empagliflozin on vascular and left ventricular myocardial function during the study period.

	All Patients (n = 54)	Dulaglutide (n = 27)	Empagliflozin (n = 27)
PBR (5–25 μm)			
Baseline	2.24 ± 0.25	2.25 ± 0.24	2.22 ± 0.25
4 months	2.13 ± 0.24 ^††^	2.11 ± 0.23 ^††^	2.14 ± 0.24
12 months	2.02 ± 0.22 ^†††^	2.01 ± 0.23 ^†††^	2.03 ± 0.21 ^†^
SBP (mmHg)			
Baseline	140.7 ± 14.3	142 ± 14.8	139.9 ± 13.8
4 months	136.9 ± 14.8 ^†^	139.2 ± 15.4 ^†^	134.5 ± 14.2 ^††^
12 months	134.8 ± 13.7 ^†††^	136.3 ± 14.6 ^††^	133.2 ± 12.8 ^††^
DBP (mmHg)			
Baseline	83.1 ± 8.7	83.9 ± 8	82.2 ± 9.4
4 months	81.9 ± 8.4	82.8 ± 7.8	80.9 ± 8.9
12 months	80.8 ± 8.2	81.4 ± 7.1	80.1 ± 9.3
Central SBP (mmHg)			
Baseline	136.5 ± 16.1	137.1 ± 15.6	135.8 ± 16.5
4 months	132.3 ± 15.1 ^†^	133.4 ± 15.2 ^†^	131.1 ± 14.9 ^†^
12 months	131.2 ± 13.8 ^†††^	132.3 ± 14.2 ^†^	130 ± 13.3 ^††^
PWV (m/s)			
Baseline	13.3 ± 3.1	13.7 ± 2.9	12.9 ± 3.2
4 months	12.3 ± 3.2 ^††^	12.9 ± 3 ^†^	11.6 ± 3.3 ^†^
12 months	11.7 ± 3.2 ^†††^	12.2 ± 2.7 ^†^	11.1 ± 3.6 ^†,^*
AIx (%)			
Baseline	20.5 (9.6–29.3)	21.2 (11.2–27.8)	19.8 (8.1–30.9)
4 months	11.9 (3.5–20.7) ^†††^	12.7 (4.2–21.2) ^†^	11.2 (2.9–20.1) ^††^
12 months	11.3 (2.3–17.9) ^†††^	11.8 (1.9–19.4) ^††^	10.9 (2.7–16.3) ^†††^
GLS (%)			
Baseline	−16.5 ± 3.3	−16.3 ± 3.1	−16.7 ± 3.4
4 months	−17.5 ± 3.2 ^†††^	−17.6 ± 3.3 ^†††^	−17.4 ± 3.1
12 months	−18.4 ± 2.9 ^†††^	−18.7 ± 2.8 ^†††^	−18.1 ± 3 ^†,^**

Data are presented as mean values ± standard deviation or median (interquartile range). Perfused boundary region of the sublingual microvessels ranged from 5 to 25 μm. PBR, perfused boundary region; SBP, systolic blood pressure; DBP, diastolic blood pressure; PWV, pulse wave velocity; AIx, augmentation index; GLS, global longitudinal strain. * *p* < 0.05, ** *p* < 0.01 for time × treatment interaction obtained by repeated measures ANOVA. ^†^ *p* < 0.05, ^††^ *p* < 0.01, ^†††^ *p* < 0.001 for comparisons of 4 or 12 months vs. baseline.

## Data Availability

The data utilized in this study is available upon reasonable request from the corresponding author.

## Abbreviations

The following abbreviations are used in this manuscript:

AIx	Augmentation Index
ANOVA	Analysis of Variance
BMI	Body Mass Index
GLP-1RA	Glucagon-Like Peptide-1 Receptor Agonists
GLS	Global Longitudinal Strain
HbA1c	Glycosylated Hemoglobin
LV	Left Ventricular
PBR	Perfused Boundary Region
PSM	Propensity Score Matching
PWV	Pulse Wave Velocity
SBP	Systolic Blood Pressure
SGLT-2i	Sodium-Glucose Cotransporter-2 Inhibitors
T2DM	Type 2 Diabetes Mellitus

## References

[1] Hadi H.A., Suwaidi J.A. (2007). Endothelial dysfunction in diabetes mellitus. Vasc. Health Risk Manag..

[2] Shi Y., Vanhoutte P.M. (2017). Macro- and microvascular endothelial dysfunction in diabetes. J. Diabetes.

[3] Yang D.R., Wang M.Y., Zhang C.L., Wang Y. (2024). Endothelial dysfunction in vascular complications of diabetes: A comprehensive review of mechanisms and implications. Front. Endocrinol..

[4] Reitsma S., Slaaf D.W., Vink H., van Zandvoort M.A., Oude Egbrink M.G. (2007). The endothelial glycocalyx: Composition, functions, and visualization. Pflügers Arch.—Eur. J. Physiol..

[5] Pries A.R., Secomb T.W., Gaehtgens P. (2000). The endothelial surface layer. Pflugers Arch..

[6] Becker B.F., Chappell D., Jacob M. (2010). Endothelial glycocalyx and coronary vascular permeability: The fringe benefit. Basic Res. Cardiol..

[7] Huxley V.H., Williams D.A. (2000). Role of a glycocalyx on coronary arteriole permeability to proteins: Evidence from enzyme treatments. Am. J. Physiol. Heart Circ. Physiol..

[8] Becker B.F., Chappell D., Bruegger D., Annecke T., Jacob M. (2010). Therapeutic strategies targeting the endothelial glycocalyx: Acute deficits, but great potential. Cardiovasc. Res..

[9] van den Berg B., Vink H. (2006). Glycocalyx perturbation: Cause or consequence of damage to the vasculature?. Am. J. Physiol. Heart Circ. Physiol..

[10] Zieman S.J., Melenovsky V., Kass D.A. (2005). Mechanisms, pathophysiology, and therapy of arterial stiffness. Arterioscler. Thromb. Vasc. Biol..

[11] Laurent S., Cockcroft J., Van Bortel L., Boutouyrie P., Giannattasio C., Hayoz D., Pannier B., Vlachopoulos C., Wilkinson I., Struijker-Boudier H. (2006). Expert consensus document on arterial stiffness: Methodological issues and clinical applications. Eur. Heart J..

[12] Safar M.E. (2008). Pulse pressure, arterial stiffness and wave reflections (augmentation index) as cardiovascular risk factors in hypertension. Ther. Adv. Cardiovasc. Dis..

[13] Schram M.T., Henry R.M., van Dijk R.A., Kostense P.J., Dekker J.M., Nijpels G., Heine R.J., Bouter L.M., Westerhof N., Stehouwer C.D. (2004). Increased central artery stiffness in impaired glucose metabolism and type 2 diabetes: The Hoorn Study. Hypertension.

[14] Stanton T., Leano R., Marwick T.H. (2009). Prediction of all-cause mortality from global longitudinal speckle strain: Comparison with ejection fraction and wall motion scoring. Circ. Cardiovasc. Imaging.

[15] Alharbi S.H. (2024). Anti-inflammatory role of glucagon-like peptide 1 receptor agonists and its clinical implications. Ther. Adv. Endocrinol. Metab..

[16] Bendotti G., Montefusco L., Pastore I., Lazzaroni E., Lunati M.E., Fiorina P. (2023). The anti-inflammatory and immunological properties of SGLT-2 inhibitors. J. Endocrinol. Investig..

[17] Ikonomidis I., Pavlidis G., Thymis J., Birba D., Kalogeris A., Kousathana F., Kountouri A., Balampanis K., Parissis J., Andreadou I. (2020). Effects of Glucagon-Like Peptide-1 Receptor Agonists, Sodium-Glucose Cotransporter-2 Inhibitors, and Their Combination on Endothelial Glycocalyx, Arterial Function, and Myocardial Work Index in Patients with Type 2 Diabetes Mellitus After 12-Month Treatment. J. Am. Heart Assoc..

[18] Ikonomidis I., Pavlidis G., Pliouta L., Katogiannis K., Maratou E., Thymis J., Michalopoulou E., Prentza V., Katsanaki E., Vlachomitros D. (2025). Effects of Glucagon-Like Peptide-1 Receptor Agonists, Sodium-Glucose Cotransporter-2 Inhibitors, and Their Combination on Neurohumoral and Mitochondrial Activation in Patients with Diabetes. J. Am. Heart Assoc..

[19] Prentza V., Pavlidis G., Ikonomidis I., Pililis S., Lampsas S., Kountouri A., Pliouta L., Korakas E., Thymis J., Palaiodimou L. (2024). Antidiabetic Treatment and Prevention of Ischemic Stroke: A Systematic Review. J. Clin. Med..

[20] Vlahu C.A., Lemkes B.A., Struijk D.G., Koopman M.G., Krediet R.T., Vink H. (2012). Damage of the endothelial glycocalyx in dialysis patients. J. Am. Soc. Nephrol..

[21] Ikonomidis I., Thymis J., Simitsis P., Koliou G.A., Katsanos S., Triantafyllou C., Kousathana F., Pavlidis G., Kountouri A., Polyzogopoulou E. (2022). Impaired Endothelial Glycocalyx Predicts Adverse Outcome in Subjects Without overt Cardiovascular Disease: A 6-Year Follow-up Study. J. Cardiovasc. Transl. Res..

[22] McEvoy J.W., McCarthy C.P., Bruno R.M., Brouwers S., Canavan M.D., Ceconi C., Christodorescu R.M., Daskalopoulou S.S., Ferro C.J., Gerdts E. (2024). 2024 ESC Guidelines for the management of elevated blood pressure and hypertension. Eur. Heart J..

[23] Sugimoto T., Dulgheru R., Bernard A., Ilardi F., Contu L., Addetia K., Caballero L., Akhaladze N., Athanassopoulos G.D., Barone D. (2017). Echocardiographic reference ranges for normal left ventricular 2D strain: Results from the EACVI NORRE study. Eur. Heart J. Cardiovasc. Imaging.

[24] Nieuwdorp M., van Haeften T.W., Gouverneur M.C., Mooij H.L., van Lieshout M.H., Levi M., Meijers J.C., Holleman F., Hoekstra J.B., Vink H. (2006). Loss of endothelial glycocalyx during acute hyperglycemia coincides with endothelial dysfunction and coagulation activation in vivo. Diabetes.

[25] Nieuwdorp M., Mooij H.L., Kroon J., Atasever B., Spaan J.A., Ince C., Holleman F., Diamant M., Heine R.J., Hoekstra J.B. (2006). Endothelial glycocalyx damage coincides with microalbuminuria in type 1 diabetes. Diabetes.

[26] Singh A., Fridén V., Dasgupta I., Foster R.R., Welsh G.I., Tooke J.E., Haraldsson B., Mathieson P.W., Satchell S.C. (2011). High glucose causes dysfunction of the human glomerular endothelial glycocalyx. Am. J. Physiol. Renal Physiol..

[27] Broekhuizen L.N., Lemkes B.A., Mooij H.L., Meuwese M.C., Verberne H., Holleman F., Schlingemann R.O., Nieuwdorp M., Stroes E.S., Vink H. (2010). Effect of sulodexide on endothelial glycocalyx and vascular permeability in patients with type 2 diabetes mellitus. Diabetologia.

[28] Perrimon N., Bernfield M. (2000). Specificities of heparan sulphate proteoglycans in developmental processes. Nature.

[29] Zuurbier C.J., Demirci C., Koeman A., Vink H., Ince C. (2005). Short-term hyperglycemia increases endothelial glycocalyx permeability and acutely decreases lineal density of capillaries with flowing red blood cells. J. Appl. Physiol..

[30] Quinsey N.S., Greedy A.L., Bottomley S.P., Whisstock J.C., Pike R.N. (2004). Antithrombin: In control of coagulation. Int. J. Biochem. Cell Biol..

[31] Del Olmo-Garcia M.I., Merino-Torres J.F. (2018). GLP-1 Receptor Agonists and Cardiovascular Disease in Patients with Type 2 Diabetes. J. Diabetes Res..

[32] Nikolaou P.E., Konijnenberg L.S.F., Kostopoulos I.V., Miliotis M., Mylonas N., Georgoulis A., Pavlidis G., Kuster C.T.A., van Reijmersdal V.P.A., Luiken T.T.J. (2024). Empagliflozin in Acute Myocardial Infarction Reduces No-Reflow and Preserves Cardiac Function by Preventing Endothelial Damage. JACC Basic Transl. Sci..

[33] Garg V., Verma S., Connelly K. (2019). Mechanistic insights regarding the role of SGLT2 inhibitors and GLP1 agonist drugs on cardiovascular disease in diabetes. Prog. Cardiovasc. Dis..

[34] Chilton R., Tikkanen I., Cannon C.P., Crowe S., Woerle H.J., Broedl U.C., Johansen O.E. (2015). Effects of empagliflozin on blood pressure and markers of arterial stiffness and vascular resistance in patients with type 2 diabetes. Diabetes Obes. Metab..

[35] Kim M., Yoo J., Baik M., Kim J., Jung I.H. (2025). Clinical Usefulness of Left Ventricular Global Longitudinal Strain as a Predictor of Prognosis in Patients with Acute Ischemic Stroke (GLS-STROKE Study). J. Am. Heart Assoc..

[36] Biering-Sørensen T., Biering-Sørensen S.R., Olsen F.J., Sengeløv M., Jørgensen P.G., Mogelvang R., Shah A.M., Jensen J.S. (2017). Global Longitudinal Strain by Echocardiography Predicts Long-Term Risk of Cardiovascular Morbidity and Mortality in a Low-Risk General Population: The Copenhagen City Heart Study. Circ. Cardiovasc. Imaging.

[37] Makavos G., Ikonomidis I., Lambadiari V., Koliou G.A., Pavlidis G., Thymis J., Rafouli-Stergiou P., Kostelli G., Katogiannis K., Stamoulis K. (2023). Additive prognostic value of longitudinal myocardial deformation to SCORE2 in psoriasis. Eur. Heart J. Open.

[38] Nikolaidis L.A., Elahi D., Hentosz T., Doverspike A., Huerbin R., Zourelias L., Stolarski C., Shen Y.T., Shannon R.P. (2004). Recombinant glucagon-like peptide-1 increases myocardial glucose uptake and improves left ventricular performance in conscious dogs with pacing-induced dilated cardiomyopathy. Circulation.

[39] Nauck M.A., Meier J.J., Cavender M.A., Abd El Aziz M., Drucker D.J. (2017). Cardiovascular Actions and Clinical Outcomes with Glucagon-Like Peptide-1 Receptor Agonists and Dipeptidyl Peptidase-4 Inhibitors. Circulation.

[40] Baartscheer A., Schumacher C.A., Wüst R.C., Fiolet J.W., Stienen G.J., Coronel R., Zuurbier C.J. (2017). Empagliflozin decreases myocardial cytoplasmic Na+ through inhibition of the cardiac Na+/H+ exchanger in rats and rabbits. Diabetologia.

[41] Santos-Gallego C.G., Requena-Ibanez J.A., San Antonio R., Ishikawa K., Watanabe S., Picatoste B., Flores E., Garcia-Ropero A., Sanz J., Hajjar R.J. (2019). Empagliflozin Ameliorates Adverse Left Ventricular Remodeling in Nondiabetic Heart Failure by Enhancing Myocardial Energetics. J. Am. Coll. Cardiol..

[42] Nielsen R., Møller N., Gormsen L.C., Tolbod L.P., Hansson N.H., Sorensen J., Harms H.J., Frøkiær J., Eiskjaer H., Jespersen N.R. (2019). Cardiovascular Effects of Treatment with the Ketone Body 3-Hydroxybutyrate in Chronic Heart Failure Patients. Circulation.

[43] Verma S., Mazer C.D., Yan A.T., Mason T., Garg V., Teoh H., Zuo F., Quan A., Farkouh M.E., Fitchett D.H. (2019). Effect of Empagliflozin on Left Ventricular Mass in Patients with Type 2 Diabetes Mellitus and Coronary Artery Disease: The EMPA-HEART CardioLink-6 Randomized Clinical Trial. Circulation.

[44] Leo I., Salerno N., Figliozzi S., Cersosimo A., Ielapi J., Stankowski K., Bisaccia G., Dellegrottaglie S., Canino G., De Rosa S. (2025). Effect of SGLT2 inhibitors on cardiac structure and function assessed by cardiac magnetic resonance: A systematic review and meta-analysis. Cardiovasc. Diabetol..

[45] Hwang I.C., Cho G.Y., Yoon Y.E., Park J.J., Park J.B., Lee S.P., Kim H.K., Kim Y.J., Sohn D.W. (2020). Different effects of SGLT2 inhibitors according to the presence and types of heart failure in type 2 diabetic patients. Cardiovasc. Diabetol..

[46] Mustapic I., Bakovic D., Susilovic-Grabovac Z., Borovac J.A. (2023). Left Ventricular Systolic Function After 3 Months of SGLT2 Inhibitor Therapy in Heart Failure Patients with Reduced Ejection Fraction. J. Cardiovasc. Transl. Res..

[47] Cheng X., Huang P., Liu H., Bi X., Gao Y., Lu R., Gao Y., Liu Y., Deng Y. (2024). Improvements of myocardial strain and work in diabetes patients with normal ejection fraction after empagliflozin treatment. J. Diabetes Investig..

[48] Mustapic I., Bakovic D., Susilovic Grabovac Z., Borovac J.A. (2022). Impact of SGLT2 Inhibitor Therapy on Right Ventricular Function in Patients with Heart Failure and Reduced Ejection Fraction. J. Clin. Med..

[49] Alcidi G., Pugliese R., Ioannoni S., Romano M., Palmieri G., Tabella E., Correale M., Di Biase M., Brunetti N.D., Iacoviello M. (2023). Improvement in Left and Right Ventricular Function after Introduction of SGLT2 Inhibitors in Heart Failure Outpatients with Reduced Ejection Fraction. Clin. Pract..

[50] Stefanou M.I., Theodorou A., Malhotra K., Aguiar de Sousa D., Katan M., Palaiodimou L., Katsanos A.H., Koutroulou I., Lambadiari V., Lemmens R. (2024). Risk of major adverse cardiovascular events and stroke associated with treatment with GLP-1 or the dual GIP/GLP-1 receptor agonist tirzepatide for type 2 diabetes: A systematic review and meta-analysis. Eur. Stroke J..

[51] Malhotra K., Katsanos A.H., Lambadiari V., Goyal N., Palaiodimou L., Kosmidou M., Krogias C., Alexandrov A.V., Tsivgoulis G. (2020). GLP-1 receptor agonists in diabetes for stroke prevention: A systematic review and meta-analysis. J. Neurol..

[52] Kleindorfer D.O., Towfighi A., Chaturvedi S., Cockroft K.M., Gutierrez J., Lombardi-Hill D., Kamel H., Kernan W.N., Kittner S.J., Leira E.C. (2021). 2021 Guideline for the Prevention of Stroke in Patients with Stroke and Transient Ischemic Attack: A Guideline From the American Heart Association/American Stroke Association. Stroke.

[53] Scheen A.J. (2023). Do SGLT2 inhibitors and GLP-1 receptor agonists modulate differently the risk of stroke? Discordance between randomised controlled trials and observational studies. Diabetes Metab..

[54] Tsai W.H., Chuang S.M., Liu S.C., Lee C.C., Chien M.N., Leung C.H., Liu S.J., Shih H.M. (2021). Effects of SGLT2 inhibitors on stroke and its subtypes in patients with type 2 diabetes: A systematic review and meta-analysis. Sci. Rep..

[55] Tuttolomondo A., Casuccio A., Della Corte V., Maida C., Pecoraro R., Di Raimondo D., Vassallo V., Simonetta I., Arnao V., Pinto A. (2017). Endothelial function and arterial stiffness indexes in subjects with acute ischemic stroke: Relationship with TOAST subtype. Atherosclerosis.

[56] Acampa M., Guideri F., Di Donato I., Tassi R., Marotta G., Lo Giudice G., D’Andrea P., Martini G. (2014). Arterial stiffness in patients with deep and lobar intracerebral hemorrhage. J. Stroke.

